# Comprehensive spectroscopic and computational insight into the binding of vanillin with human transferrin: targeting neuroinflammation in Alzheimer’s disease therapeutics

**DOI:** 10.3389/fphar.2024.1397332

**Published:** 2024-05-10

**Authors:** Mohammed Alrouji, Sabina Yasmin, Fahad A. Alhumaydhi, Sharaf E. Sharaf, Moyad Shahwan, Mohammad Furkan, Rizwan Hasan Khan, Anas Shamsi

**Affiliations:** ^1^ Department of Medical Laboratories, College of Applied Medical Sciences, Shaqra University, Shaqra, Saudi Arabia; ^2^ Department of Pharmaceutical Chemistry, College of Pharmacy, King Khalid University, Abha, Saudi Arabia; ^3^ Department of Medical Laboratories, College of Applied Medical Sciences, Qassim University, Buraydah, Saudi Arabia; ^4^ Pharmaceutical Sciences Department, College of Pharmacy, Umm Al-Qura University, Makkah, Saudi Arabia; ^5^ Department of Clinical Sciences, College of Pharmacy and Health Sciences, Ajman University, Ajman, United Arab Emirates; ^6^ Center of Medical and Bio-Allied Health Sciences Research (CMBHSR), Ajman University, Ajman, United Arab Emirates; ^7^ Department of Biochemistry, Aligarh Muslim University, Aligarh, India; ^8^ Interdisciplinary Biotechnology Unit, Aligarh Muslim University, Aligarh, India

**Keywords:** oxidative stress, molecular dynamic simulation, fluorescence spectroscopy, Alzheimer's disease, vanillin

## Abstract

In present times, vanillin stands out as a promising therapeutic molecule that can be implicated in the treatment of neurodegenerative disorders (NDs), notably Alzheimer’s disease (AD). This can be attributed to the highly potent scavenging activity of vanillin against reactive oxygen species (ROS). Oxidative stress leads to generation of ROS that serves a critical role in AD’s pathological progression. It is apparent from various studies that diets rich in polyphenols prevent oxidative stress associated with AD development, implying the crucial role of vanillin in AD therapeutics. It is crucial to maintain iron balance to manage AD associated oxidative stress, unveiling the significance of human transferrin (hTf) that maintains iron homeostasis. Here, we have performed an integrated study of spectroscopic and computational approaches to get insight into the binding mechanism of vanillin with hTf. In the preliminary study, molecular docking deciphered that vanillin primarily occupies the hTf binding pocket, forming multiple interactions with its key residues. Moreover, the binding mechanism was evaluated at an atomistic level employing comprehensive molecular dynamic (MD) simulation. MD analysis demonstrated that binding of vanillin to hTf stabilizes its structure, without inducing any significant alterations in its native conformation. The docked complex was maintained throughout the simulations without changing its original conformation. Essential dynamics analysis further confirms that hTf achieved a stable conformation with vanillin. The outcomes were further supplemented by fluorescence spectroscopy which confirms the formation of stable hTf-vanillin complex. Taken together, the current study unveils the interaction mechanism of vanillin with hTf and providing a platform to use vanillin in AD therapeutics in the context of iron homeostasis.

## 1 Introduction

Presently, bioactive natural products are vital in drug development due to their favorable safety profile and potent antioxidant capabilities along with their broad therapeutic potentials and minimal side effects. All these characteristics make them a vital cog in disease therapeutics, highlighting their importance in the current disease era. Vanillin, a natural phenolic compound plentiful in various vanilla beans, is extensively employed in the food, cosmetic, and pharmaceutical industries (Arya et al., 2021). Apart from its industrial usage, vanillin offers several health benefits, namely, antioxidant properties, anti-inflammatory, anti-mutagenic, anti-metastatic, and anti-depressant effects (Bezerra-Filho et al., 2019; Costantini et al., 2021). Vanillin serves as the principal and highly stable curcumin degradation product, a natural polyphenol (Wang et al., 1997). In recent times, studies have reported neuroprotective and anticancer effect of vanillin, implying its therapeutic potential (Li et al., 2018; Rakoczy et al., 2021; Yousuf et al., 2021; Iannuzzi et al., 2023). Neuroinflammation stands out as a prevalent pathological process in various neurological diseases, intricately linked to the fundamental mechanisms of neurodegenerative disorders (NDs). The primary hallmarks of neurodegeneration are Neuroinflammation and oxidative stress (Singh et al., 2019; Teleanu et al., 2022). Alzheimer’s disease (AD), an age-related ND, is a complex multifactorial disease (Lane et al., 2018). The primary neuropathological indicators of AD encompass the development of “neurofibrillary tangles” (NFTs) and accumulation of “amyloid beta” (Aβ) plaques within the brain (Iannuzzi et al., 2023). Additionally, cholinergic deficit, along with oxidative stress and neuroinflammation, plays a vital role in processes that lead to neuronal death (Butterfield and Halliwell, 2019; Guo et al., 2020). A recent study established that vanillin decreased the Aβ effects on learning and memory by suppressing oxidative stress. Another research stated that vanillin showed an acetylcholinesterase inhibitory activity *in vitro*, implying vanillin to be safe and effective natural drug candidate offering enormous promise for AD management.

Presently, the precise cause of AD remains poorly understood with age recognized as the primary risk factor for AD (Imtiaz et al., 2014). The major problem associated with AD therapeutics is that no disease-modifying drugs are currently available and moreover, clinical trials have highest failure rates in any therapeutic area (Yiannopoulou and Papageorgiou, 2013; Chen and Pan, 2015). Oxidative stress plays a vital role in the pathological progression of AD, along with various other diseases such as cancer, diabetes, and neurological disorders (Markesbery, 1997). Many studies have reported that consuming diets abundant in polyphenols prevent the oxidative stress associated with the AD pathogenesis (Kundu and Mitra, 2013; Cremonini et al., 2019; Salau et al., 2020; Ahammed et al., 2021). Various studies have reported vanillin’s anti-inflammatory effects and its anti-oxidant activity (Zhao et al., 2017; Gu et al., 2019). Given its strong scavenging activity against reactive oxygen species (ROS) and its ability to inhibit inflammatory pathway activation, vanillin emerges as a promising therapeutic molecule for the prevention of AD and other NDs. Studies have reported that vanillin shows “AChE inhibitory activity”, *in vitro* and *in vivo*, hinders amyloid aggregation, and provides protection against neuronal oxidative damage (Ahammed et al., 2021). Vanillin has been reported to not only inhibit AChE activity but also restore oxidative balance in Fe^2+−^induced brain tissue damage (Salau et al., 2020). All these studies highlight the importance of vanillin in providing protection against neuronal oxidative damage observed in AD and other NDs conditions.

Free iron serves as a potent neurotoxin capable of triggering the generation of ROS, creating an environment of oxidative stress. In its unbound state, iron functions as a formidable neurotoxin, possessing the potential to induce oxidative stress (Shahwan et al., 2023). The production of ROS and Reactive nitrogen species (RNS), directly implicated in the inflammatory process, can markedly impact iron metabolism by interacting with iron-regulatory proteins (IRPs). Thus, maintaining proper iron levels is vital and human transferrin (hTf) plays a key role in iron homeostasis along with ferritin. Iron homeostasis plays a vital role in AD associated oxidative stress, thus implying the significant role played by hTf in AD therapeutics. Owing to pivotal role of vanillin and hTf, the present study encompasses a combination of experimental and computational approaches to delineate the atomistic insights into the binding of vanillin with hTf.

## 2 Materials and Methods

### 2.1 Materials

Human transferrin (hTf) and vanillin were bought from Sigma Aldrich (St. Louis, USA). For buffer preparations, all the chemicals were of the best quality purchased from HiMedia. All the buffers were filtered before use and for spectroscopic assays, corresponding blanks were taken.

### 2.2 Molecular docking

Molecular docking studies were conducted on a DELL^®^ workstation to elucidate the interactions between Vanillin and hTf. Computational tools were used to optimize visualization, evaluation, and analysis of the docking results. We obtained three-dimensional configuration of hTf (PDB ID: 3V83) and PubChem database (PubChem CID: 1183) was used to obtain vanillin structure. We prepared vanillin structure using MGL AutoDock tools (Huey et al., 2012). Further, docking simulations were carried out using AutoDock Vina (Trott and Olson, 2010). We employed PLIP to explore the intricate ligand-receptor interactions between vanillin and hTf (Salentin et al., 2015). To gain an in-depth understanding of the binding conformation and the nature of interactions between vanillin and hTf, PyMOL (DeLano, 2002) and Discovery Studio Visualizer (Biovia, 2017) were used.

### 2.3 MD simulations

MD simulations were conducted to explore the dynamic behavior of hTf and hTf-vanillin complex. We performed a simulation at 300 K temperature, using charmm36-jul2022 force field (Huang and MacKerell, 2013), implemented within GROMACS version 2020 (Van Der Spoel et al., 2005). The topology for vanillin was generated using CHARMM CGenFF program, using a Python script. hTf and hTf-Vanillin were later solvated in cubic boxes with 1 nm dimensions in the SPC216 water model using *GROMACS gmx solvate* tool (Glättli et al., 2002). Both systems were neutralized by adding suitable quantities of counterions to achieve a concentration of 0.15 M. Equilibration processes in the NVT and NPT ensembles were conducted, where the systems were slowly heated to 300 K over 1000 ps. Subsequently, solvent molecules were allowed to relax in the NPT ensemble for an additional 1000 ps without any restraints, while maintaining a pressure of 1 bar using the Berendsen Barostat method. To constrain bond lengths, the LINCS algorithm was applied, while SETTLE restrained water molecules. The system’s energy was minimized with 1500 iterations of the steepest descent approach. We employed a range of tools provided by GROMACS to analyze trajectories generated during MD simulations. The results obtained from the MD simulations were presented graphically using the XMGrace software (Turner, 2005). Graphs and figures were created to illustrate key findings, aiding in a clear and concise explanation of the obtained results.

### 2.4 Principal component analysis and free energy landscapes

Principal component analysis (PCA) is an essential tool for understanding the primary motions and conformational sampling of proteins in MD simulations. The MD trajectories of hTf and hTf-vanillin underwent PCA to explore their predominant modes of motion. This involved diagonalizing the covariance matrix to derive the first two eigenvectors (EVs), which depict the primary motions of the protein or protein-ligand complex. Additionally, we generated free energy landscapes (FELs) to assess the stability and folding dynamics of hTf and its complex with vanillin. Conformational sampling techniques were employed to construct FELs, enabling the visualization of energy landscapes and the identification of stable conformations or metastable states.

### 2.5 Fluorescence spectroscopy

We performed fluorescence spectroscopy on Shimadzu (RF 6000) spectrofluorometer. The concentration of hTf was fixed at 4 µM and vanillin was varied in a range of “0–5 µM”. The quenching data was analyzed using “Modified Stern–Volmer” equation (MSV) to compute binding parameters of hTf-vanillin complex.

## 3 Result and discussion

### 3.1 Molecular docking

**FIGURE 1:**
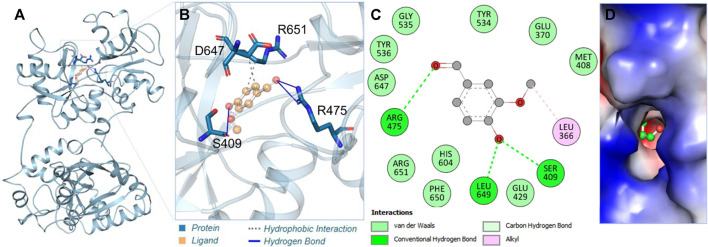
Interaction analysis. **(A)** Illustration of vanillin binding with hTf. **(B)** Ribbon representation depicting the docking of vanillin. **(C)** Two-dimensional interaction plot illustrating vanillin’s binding interactions with hTf. **(D)** Visualization of the surface potential in the hTf binding pocket occupied by vanillin.

Vanillin showed remarkable interactions towards hTf, resulting in a highly favorable binding affinity in triplicate dodcking experiments. Vanillin binds to hTf with an average binding affinity of −5.8 kcal/mol, implying vanillin’s potential as a potent hTf binding partner. Vanillin is predicted to primarily interact with the hTf binding pocket (Figure 1A). Notably, vanillin forms three hydrogen bonds with key residues, *viz.* Ser409, Arg475, and Leu649 (Figure 1B; Supplementary Table S1). Tyr536 is a vital active site residue that plays a key role for iron (Fe^3+^) binding in hTf, which plays a key role in forming these interactions (Noinaj et al., 2012). Tyr536 forms a hydrophobic bond with vanillin, further stabilizing the complex (Figure 1B). The 2D and surface plots further shed light on other interactions playing a key role in stabilizing the hTf-vanillin complex. Arg475 is another active site residuewhich is responsible to bind with hydrogencarbonate. The surface representation of the docked complex further revealed that vanillin fits into the internal cavity of hTf (Figures 1C, D). Earlier studies have also reported iron binding to hTf showing similar interactions, thereby validating our findings (Noinaj et al., 2012; Ghanbari et al., 2017). Overall, it is indicated that vanillin binds to the hTf binding pocket.

### 3.2 MD simulation analysis

**FIGURE 2:**
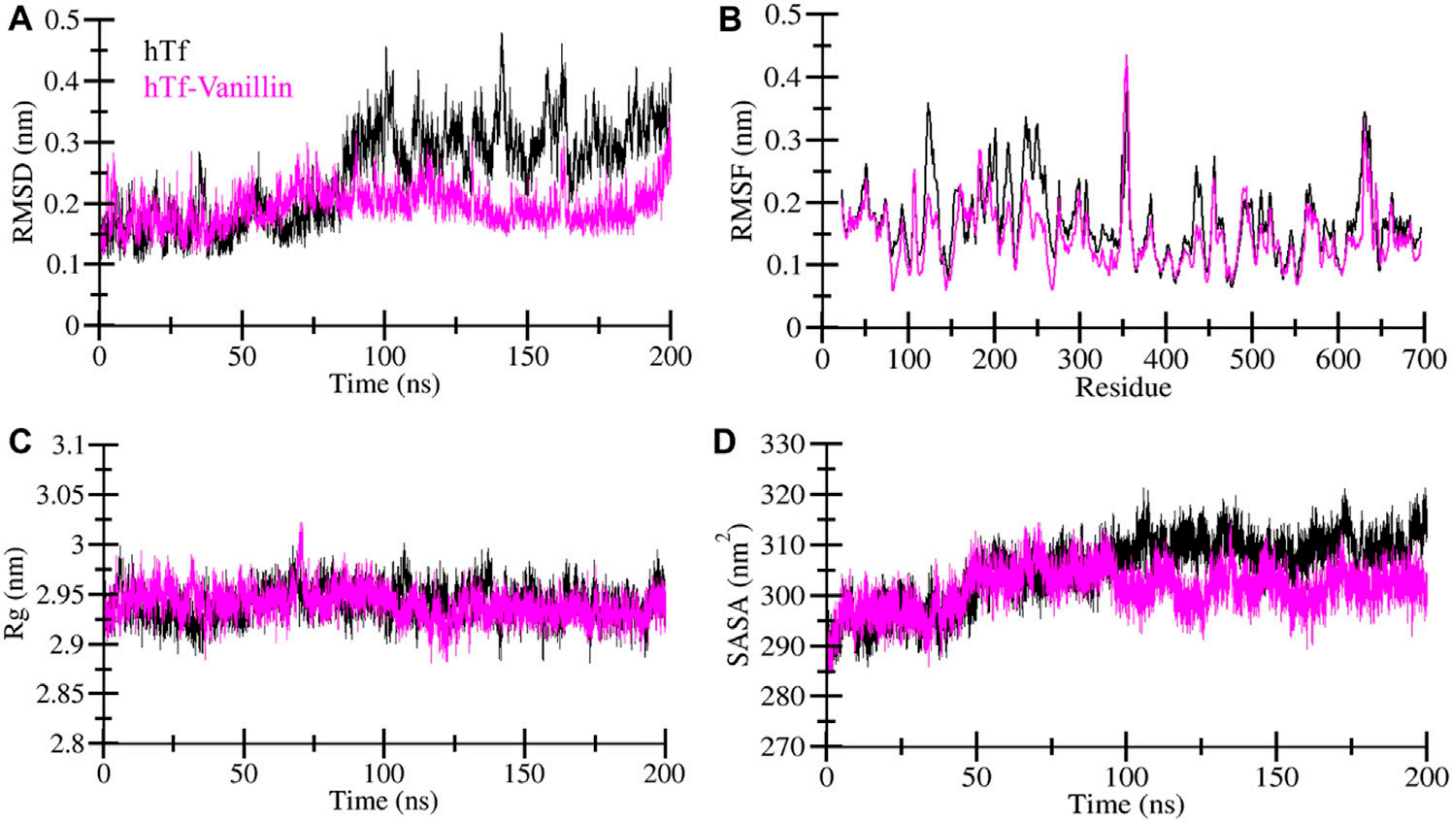
Exploring the structural dynamics of hTf in the presence of vanillin **(A)** RMSD **(B)** RMSF **(C)** Rg, and **(D)** SASA plots.

MD simulations are a widely adopted computational technique for unraveling the underlying dynamics and structural alterations of proteins and protein-ligand complexes (Shukla and Tripathi, 2020). MD simulation serves as a valuable tool for probing the biomolecular interactions within a defined time frame (Mohammad et al., 2020). In this specific investigation, we conducted a comprehensive MD simulation spanning 200 nanoseconds, encompassing hTf and the hTf-vanillin docked complex. Our key goal was to scrutinize changes in conformation, assess stability, and unravel the fundamental mechanisms governing the interaction between vanillin and hTf. First, to evaluate the structural changes, we employed the root mean square deviation (RMSD) on the simulated trajectory. Our findings reveal that vanillin binding leads to the stabilization of hTf without inducing any significant alterations in its inherent configuration, as clearly depicted in Figure 2A. However, during the initial phase, the RMSD showed intermittent fluctuations, primarily attributable to the accommodation of vanillin within hTf’s binding site.

To delve into local structural changes and residue flexibility, we calculated and visually represented the root-mean-square fluctuation (RMSF) of the simulated systems (Figure 2B). The RMSF plot highlights varying levels of residue fluctuations across different zones of the hTf structure. Intriguingly, the overall average RMSF of hTf and hTf-vanillin exhibited a consistent pattern. Importantly, the introduction of vanillin resulted in heightened residue fluctuations, indicative of increased system dynamics. These enhanced fluctuations may be attributed to residual vibrations during the simulation process.

The radius of gyration (*R*g) is a commonly utilized measure to glean insights into the folding and structural conformation of the proteins. A higher *R*g typically implies a less condensed conformation. In the case of the hTf-vanillin, our observations indicate a minor decrease in *R*g compared to the unbound hTf, signifying that the structure maintains compactness throughout the simulation, as illustrated in Figure 2C. Despite slight variations in *R*g, there are no significant alterations in the packing of hTf following the binding of vanillin. These findings underscore that vanillin induces minimal structural deviations in hTf.

The solvent-accessible surface area (SASA) represents the fraction of a protein’s surface directly exposed to the adjacent solvent and is closely associated with the *R*g. We calculated and visually depicted the average SASA for hTf and hTf-vanillin from the simulated trajectory. In the instance of the hTf-vanillin complex, we observed a minor decrease in SASA compared to the free hTf, implying the maintenance of structural compactness during the simulation. Despite minimal variations in SASA, no significant alterations in the packing of hTf were discernible following the binding of vanillin (Figure 2D).

**FIGURE 3:**
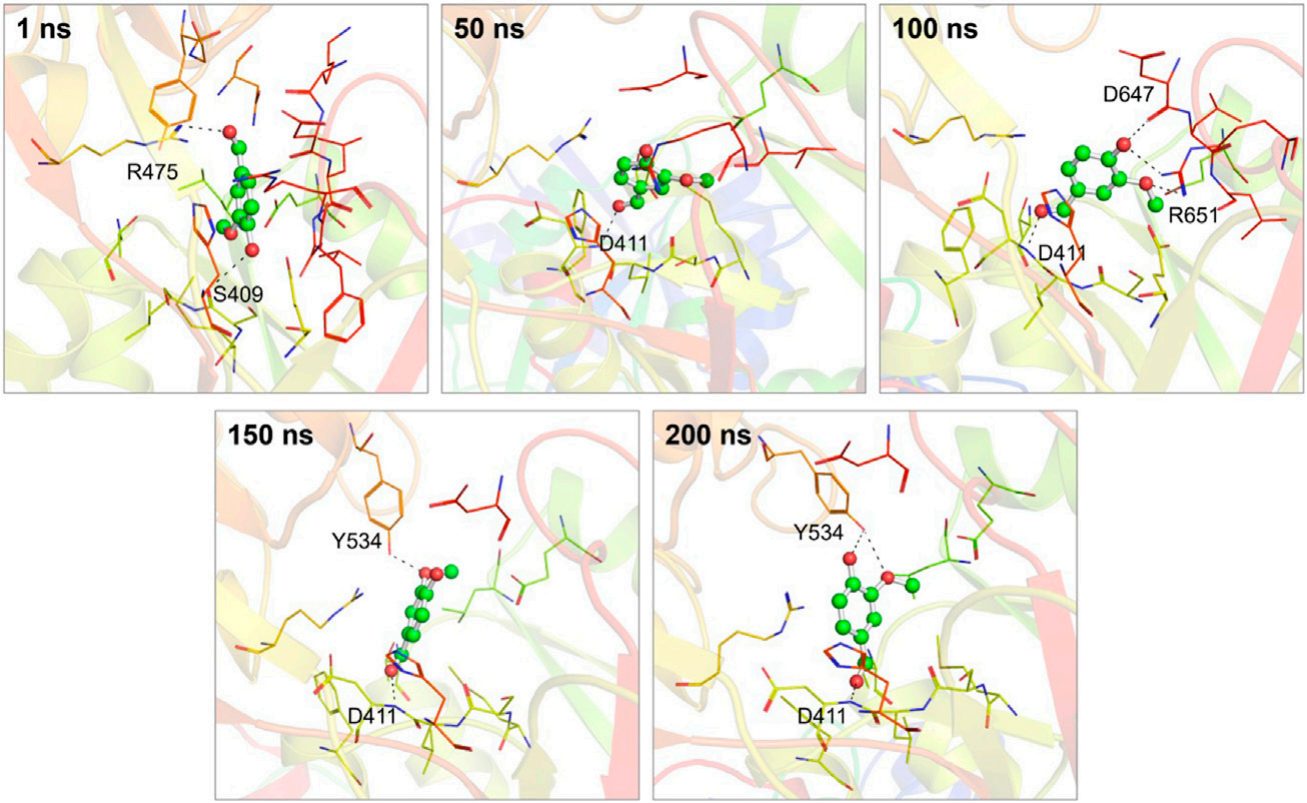
Conformational dynamics of the hTf-vanillin complex at different time intervals during 200 ns. The binding conformations showing hTf-vanillin interactions at 1, 50, 100, 150, and 200 ns, respectively.

Moreover, by employing MD simulation trajectories, we have successfully depicted the conformational snapshots showcasing the binding interactions between vanillin and hTf. This analysis enabled us to monitor the temporal evolution of the binding prototype. Structural assessments were performed at five distinct time intervals: 1, 50, 100, 150, and 200 ns, as illustrated in Figure 3. Notably, our investigation unveiled that vanillin maintained its initial docking positions consistently throughout the simulation, indicating minimal conformational alterations.

### 3.3 Stabilization of hTf-Vanillin complex

**FIGURE 4:**
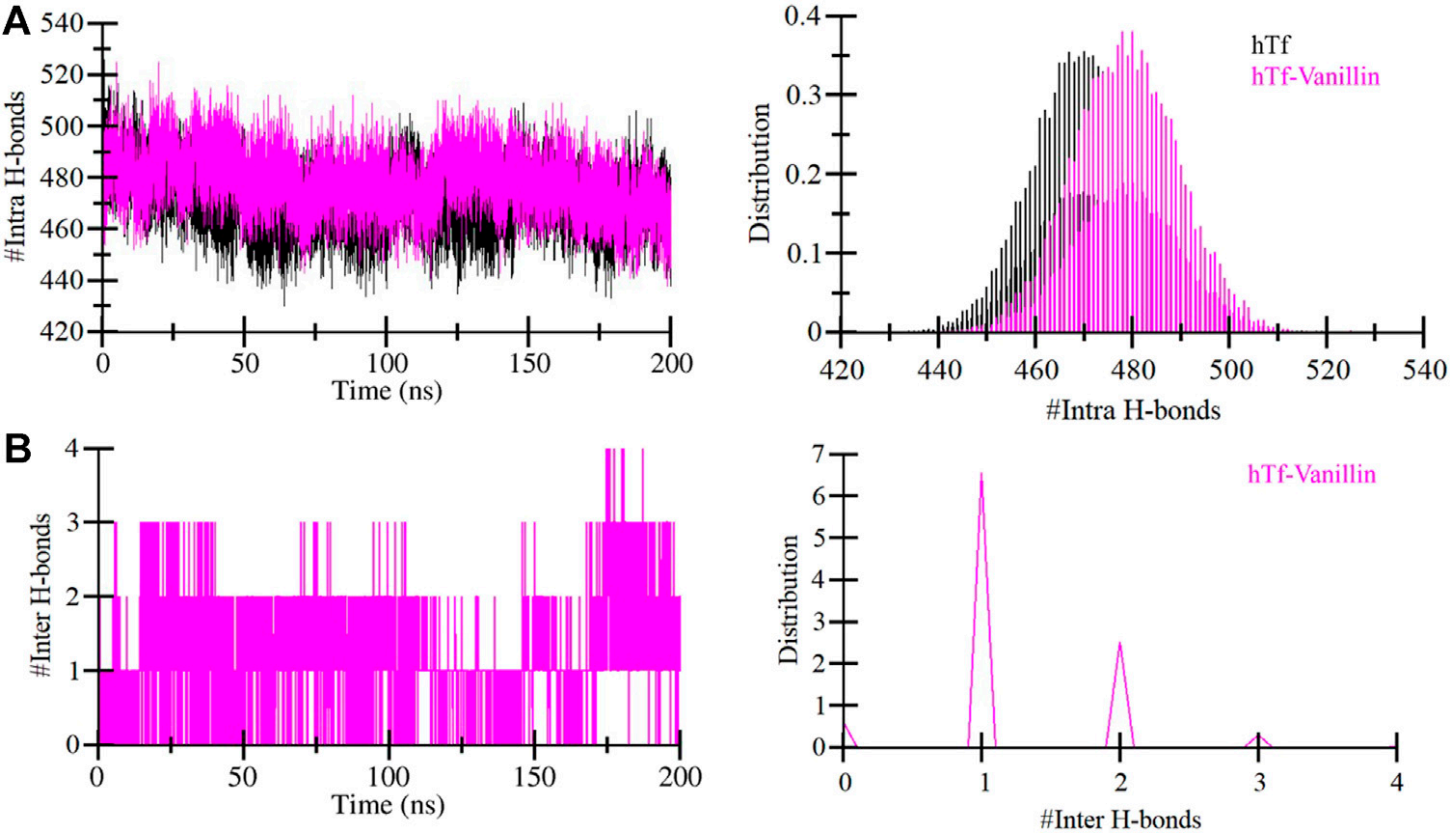
Temporal development of hydrogen bonds established **(A)** Within hTf, and **(B)** Hydrogen bonds between vanillin and hTf.

Evaluating the dynamics of hydrogen bonding is crucial for assessing the stability of the proteins and protein-ligand complexes. In this investigation, we quantified and explored the dynamics of hydrogen bonds intramolecularly within hTf and intermolecularly between hTf and vanillin (Figure 4). Upon the vanillin binding to hTf, we observed a minor increase in the number of intramolecular hydrogen bonds within hTf due to the higher compactness of the protein (Figure 4A). We also calculated the intermolecular hydrogen bonds formed between hTf and vanillin. Our analysis revealed the presence of up to 4 hydrogen bonds within the protein-ligand complex. We also observed that vanillin effectively nestled within hTf’s iron-binding pocket, forming 3-4 hydrogen bonds with some fluctuation and 1-2 hydrogen bonds with higher stability (Figure 4B). This thorough analysis provides additional supportive evidence for the stable binding of hTf and vanillin.

### 3.4 Principal component analysis

**FIGURE 5:**
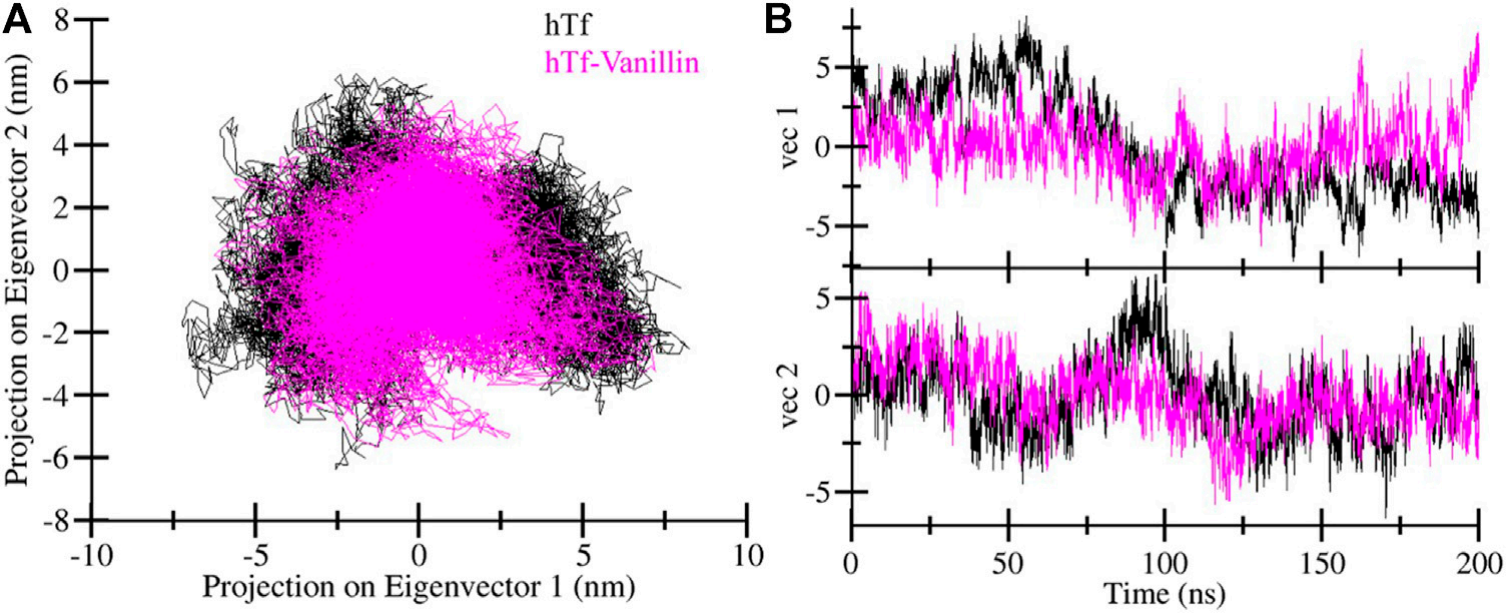
Principal component analysis. **(A)** Two-dimensional projections of hTf conformation, and **(B)** Temporal evolution of trajectory projections.

Principal component analysis (PCA) offers valuable insights into protein stability and conformational landscape (Stein et al., 2006). Here, we employed PCA to investigate the conformational dynamics of hTf and hTf-vanillin from the simulated trajectory. Figure 5 demonstrates the conformational dynamics of these systems along eigenvector 1 and eigenvector 2. The PCA plot indicates that in the presence of vanillin, hTf explores a more confined range of phase spaces (Figure 5A). We did not observe any significant overarching transitions or major conformational changes in the motion of hTf following vanillin binding (Figure 5B). This implies that the vanillin binding does not cause substantial conformational changes in hTf.

### 3.5 Free energy landscape analysis

**FIGURE 6:**
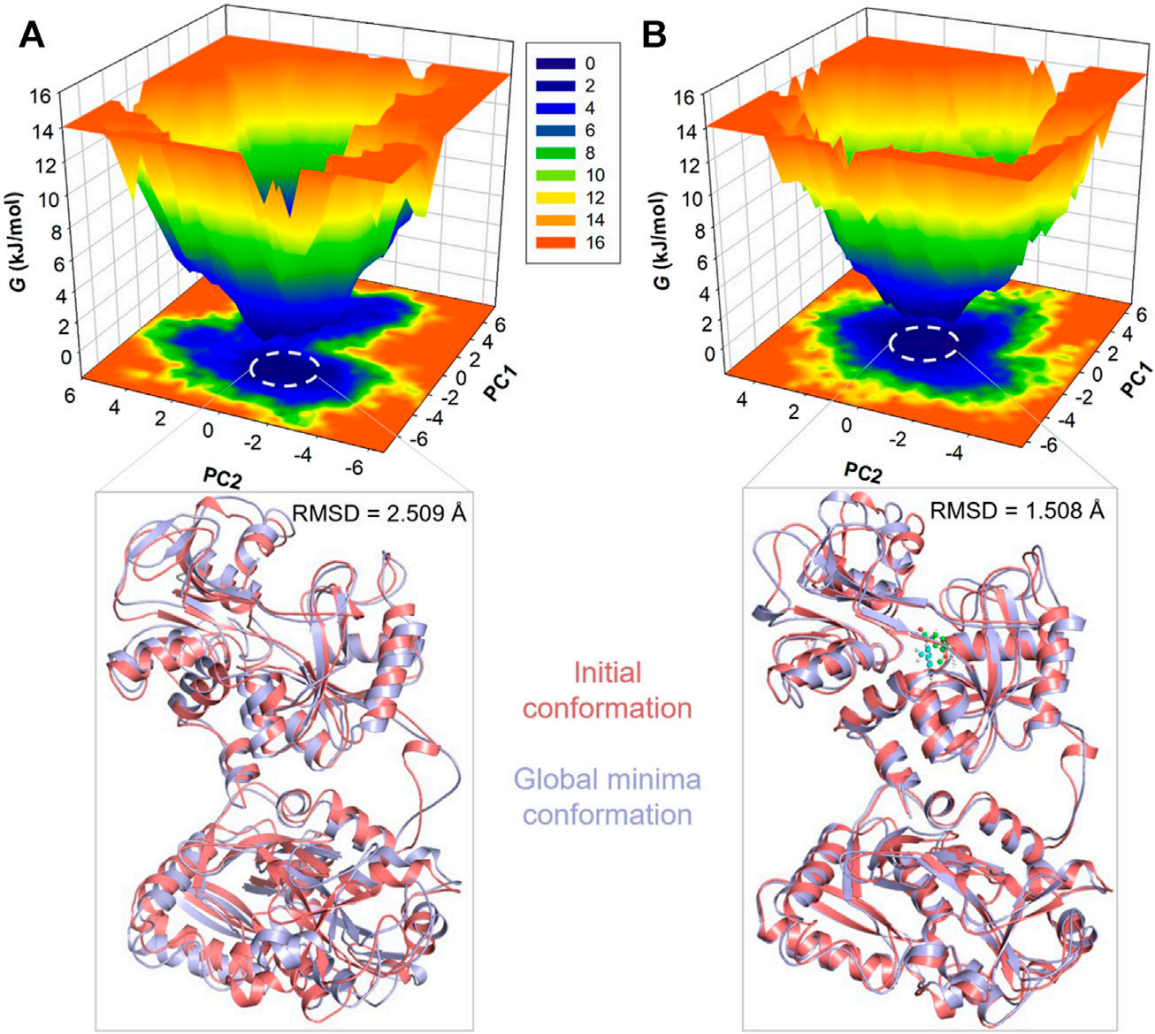
Free energy landscape plots for **(A)** Unbound hTf and **(B)** The hTf-Vanillin complex. Lower panels showed the global minima conformations of hTf in comparison to the initial conformations.

To gain deeper insights into the folding landscape of the systems, we delved into the free energy landscapes (FELs) generated through the simulated trajectories. Figure 6 presents a visual representation of the FELs for hTf and hTf-vanillin. In hTf’s FEL, it is indicated that hTf explores two distinct energy minima confined within 2-3 basins (Figure 6A). Conversely, when vanillin is introduced, a noticeable feature is a prominent single global minimum, mainly concentrated within a single basin, as illustrated in Figure 6B. Importantly, no significant or drastic conformational rearrangements are observed due to this binding event. This observation implies that vanillin binding introduces novel conformational states and influences hTf’s energy landscape. Examining these FELs provides valuable details about the conformational stability of hTf and hTf-vanillin.

### 3.6 Fluorescence binding

**FIGURE 7:**
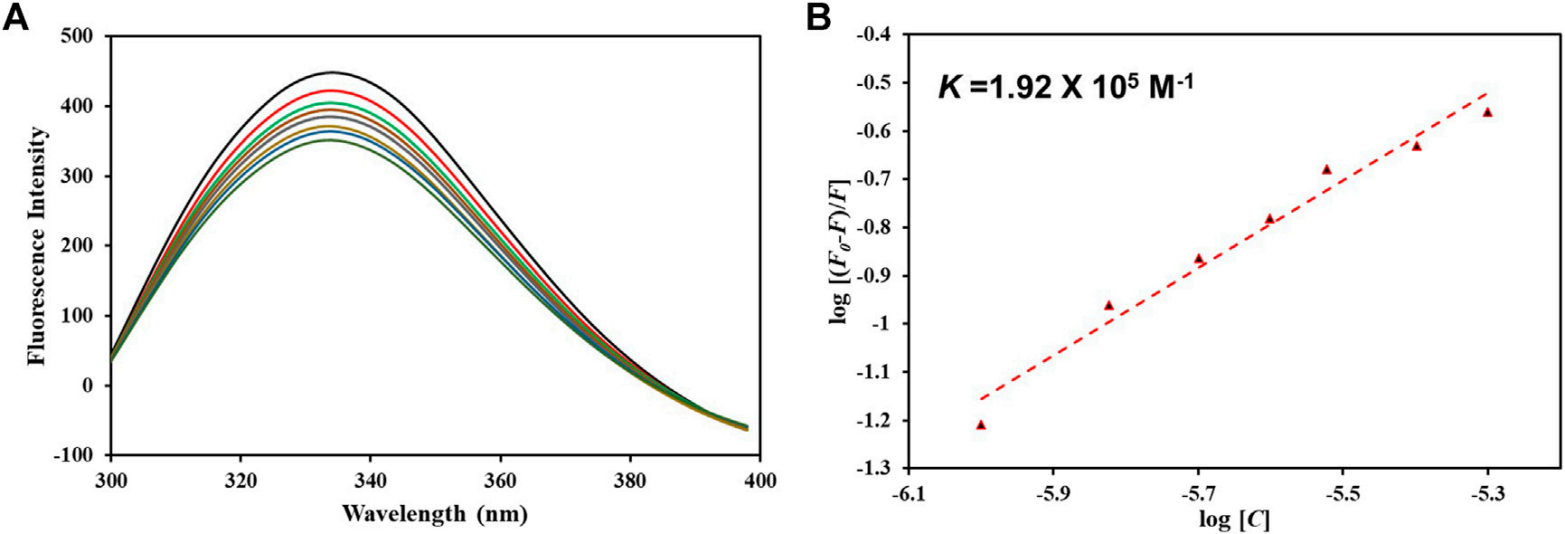
**(A)** Fluorescence emission spectra of free hTf and hTf with different vanillin concentrations (0–5 µM). **(B)** MSV plot of hTf-vanillin interaction.

After confirming hTf-vanillin complex formation through computational approaches, we used fluorescence binding to validate the obtained results. It is a powerful technique to study protein-ligand interactions, ascertaining the actual affinity of a ligand with the protein. Figure 7A shows the fluorescence emission spectra of hTf without vanillin and in the presence of varying vanillin concentrations (0–5 µM). It is apparent that with increasing vanillin concentrations, a decrease in the fluorescence intensity of hTf was apparent. The obtained quenching data was fitted into MSV equation as per earlier reports (Shamsi et al., 2020) with intercept of the obtained MSV plot (Figure 7B) giving the binding constant (*K*) of hTf-vanillin complex. Vanillin binds to hTf with a significant affinity forming a stable hTf-vanillin complex; evident from the obtained *K* of 1.92 × 10^5^ M^-1^, suggestive of strong binding between vanillin and hTf.

## 4 Conclusion

Collectively, this investigation implies that vanillin possesses the capability to impede hTf’s function through strong and persistent interactions, predominantly characterized by hydrogen bonds with crucial amino acids. Our molecular docking simulations unveiled strong and favorable interactions between vanillin and hTf, with vanillin predominantly occupying the hTf binding pocket. Notably, vanillin formed multiple hydrogen bonds with key residues of hTf, indicative of stable binding. MD simulations further corroborated these findings by demonstrating that the binding of vanillin to hTf stabilizes the protein structure without inducing significant conformational alterations. Analysis of hydrogen bonding dynamics, radius of gyration, and solvent-accessible surface area supported the stability of the hTf-vanillin complex throughout the simulation. Furthermore, fluorescence spectroscopy provided experimental validation of the hTf-vanillin interaction, confirming the formation of a stable complex with significant binding affinity. The blend of molecular docking, MD simulations, and diverse analytical approaches along with spectroscopic approach yields a holistic comprehension of the interaction between hTf and vanillin. While our study contributes valuable insights into the potential therapeutic role of vanillin in AD by targeting iron homeostasis through hTf binding, it is not without limitations. Conducting longer simulations, *in vivo* studies, elucidating high-resolution structures, and exploring combination therapies represent promising avenues for advancing the development of vanillin-based interventions for AD and other NDs.

## Data Availability

The original contributions presented in the study are included in the article/Supplementary Material, further inquiries can be directed to the corresponding author.
