# Supplementary material for: Comprehensive spectroscopic and computational insight into the binding of vanillin with human transferrin: targeting neuroinflammation in Alzheimer’s disease therapeutics
Source: Front Pharmacol. 2024 May 10;15:1397332. doi: 10.3389/fphar.2024.1397332 (PMC11116798; doi:10.3389/fphar.2024.1397332)
Supplement: Supplementary file 1 [file Presentation1.PPTX]

## Slide 1
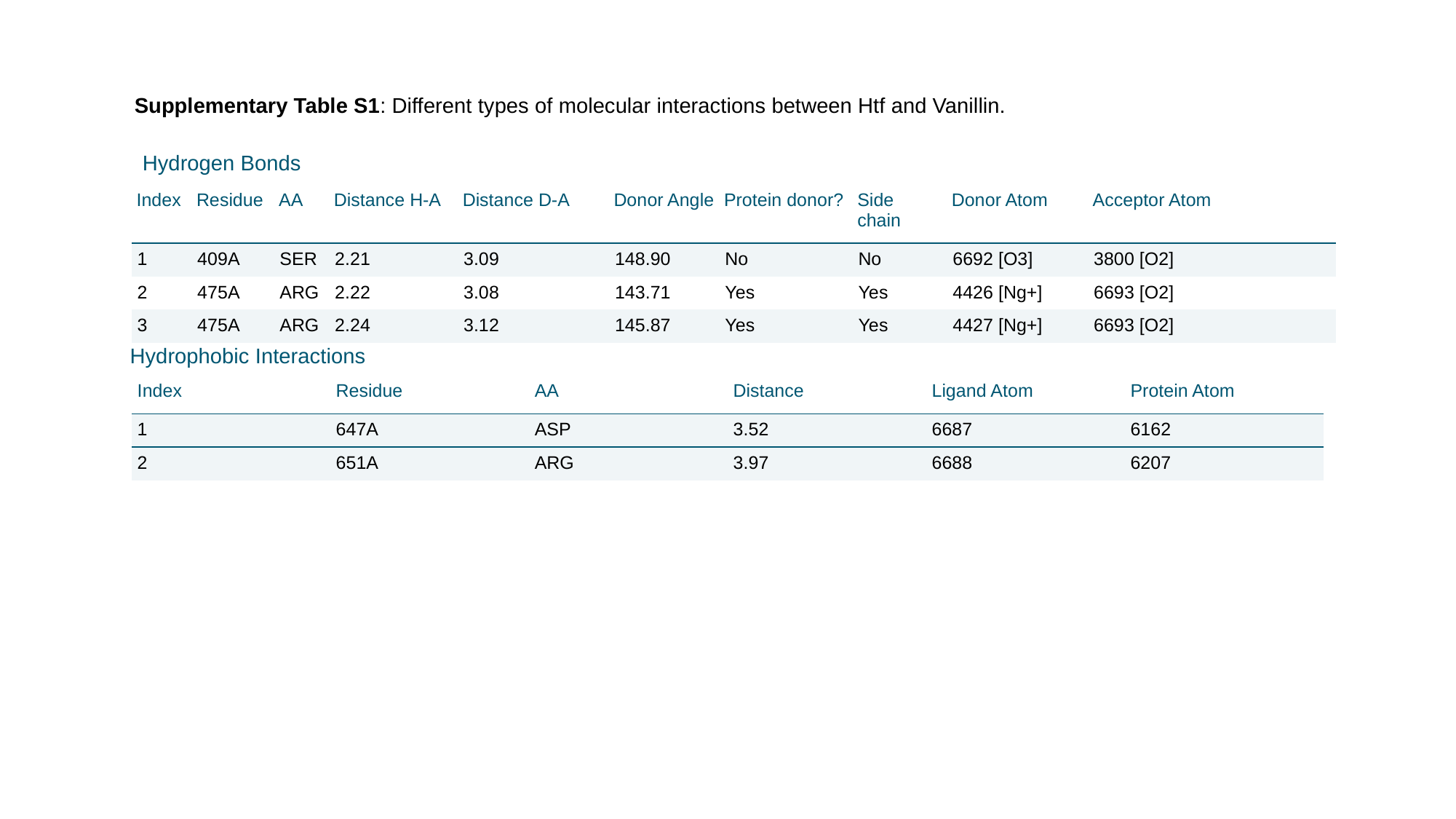

Supplementary Table S1: Different types of molecular interactions between Htf and Vanillin.
Hydrogen Bonds
| Index | Residue | AA | Distance H-A | Distance D-A | Donor Angle | Protein donor? | Side chain | Donor Atom | Acceptor Atom |
| --- | --- | --- | --- | --- | --- | --- | --- | --- | --- |
| 1 | 409A | SER | 2.21 | 3.09 | 148.90 | No | No | 6692 [O3] | 3800 [O2] |
| 2 | 475A | ARG | 2.22 | 3.08 | 143.71 | Yes | Yes | 4426 [Ng+] | 6693 [O2] |
| 3 | 475A | ARG | 2.24 | 3.12 | 145.87 | Yes | Yes | 4427 [Ng+] | 6693 [O2] |
Hydrophobic Interactions
| Index | Residue | AA | Distance | Ligand Atom | Protein Atom |
| --- | --- | --- | --- | --- | --- |
| 1 | 647A | ASP | 3.52 | 6687 | 6162 |
| 2 | 651A | ARG | 3.97 | 6688 | 6207 |
